# Case of Ulcerated Stomach, Causing Death by Being Suddenly Detached from Its Adhesions to the Peritoneal Lining of the Abdomen

**Published:** 1845-11

**Authors:** William Collins

**Affiliations:** Surgeon, Kenton


*Case of Ulcerated Stomach, Causing Death by being Suddenly Detached from its Adhesion to the Peritoneal Lining of the Abdomen.* By William Collins, Esq., Surgeon, Kenton.—I was requested by a friend to open the body of a female relative, who had died suddenly under circumstances which he could not satisfactorily account for, and I extract from my case-book the following particulars which he gave me, and the result of the autopsy.

E. W., aged 21, a very fine young woman, inclined to be corpulent, with a florid complexion, robust, and of active habits, had occasionally complained of rather acute pain in the left hypochondrium, after taking a full meal, but as it never lasted an hour at a time, and as her digestion was good, nothing was prescribed for her but some aperient pills. Being corpulent she was accustomed to have her stays laced very tight, and used to wear also a broad band round her waist, which was always made excessively tight, and it was thought the pressure might have occasioned the pain. One morning, after having used great exertion dancing all night at a ball, she eat a hearty breakfast, and quickly after walked out with some young friends. Suddenly she was seized with very severe pain in her left side, from which she said there was something tearing away; she shrieked violently, became faint, and fell down in the street; she was immediately removed to the house of her relative, which she had just left, efficient medical aid was instantly obtained, but after suffering intense agony she expired.

Twelve hours after death I opened the abdominal cavity, there did not appear to be any omentum, but merely a ragged sort of fringe along the greater curvature of the stomach; there was no appearance of inflammation on the outer coat of the stomach or intestines; but on that portion of the lesser curvature of the stomach anteriorly, which in the erect position of the body would have been in contact with the abdominal parietes on the left side, there was a considerable deposit of coagulable lymph, and a perforation through the coat of the stomach from internal ulceration, there being also a corresponding deposit of lymph on the membranous lining of the abdominal cavity, to which it was apparent the stomach had adhered, and it was opposite this spot she had always complained of pain. The stomach was very large, and contained a good deal of undigested food, but the fluid parts had escaped into the cavity of the abdomen. All the other abdominal viscera were perfectly sound.

The stomach was produced for inspection.—*Prov. Med. Jour.*

